# Structure-activity relationship optimization for lassa virus fusion inhibitors targeting the transmembrane domain of GP2

**DOI:** 10.1007/s13238-018-0604-x

**Published:** 2019-01-10

**Authors:** Guangshun Zhang, Junyuan Cao, Yan Cai, Yang Liu, Yanli Li, Peilin Wang, Jiao Guo, Xiaoying Jia, Mengmeng Zhang, Gengfu Xiao, Yu Guo, Wei Wang

**Affiliations:** 1 State Key Laboratory of Virology, Wuhan Institute of Virology, Chinese Academy of Sciences, Wuhan, 430071 China; 2 College of Pharmacy and State Key Laboratory of Medicinal Chemical Biology, Nankai University, Tianjin, 300450 China; 3 University of the Chinese Academy of Sciences, Beijing, 100049 China; 4 Drug Discovery Center for Infectious Disease, Nankai University, Tianjin, 300071 China; 5 Tianjin Key Laboratory of Molecular Drug Research, Tianjin International Joint Academy of Biomedicine, Tianjin, 300450 China

**Dear Editor,**

Lassa virus (LASV) belongs to the *Mammarenavirus* genus, *Arenaviridae* family. Arenaviruses are classified into two main groups—Old World (OW) and New World (NW)—based on virus genetics, serology, antigenic properties and geographical relationships. The OW LASV and Lujo virus (LUJV), as well as NW Junín virus (JUNV), Machupo virus (MACV), Guanarito virus (GTOV), Sabiá virus (SABV) and Chapare virus (CHAPV), are known to cause severe hemorrhagic fever and are listed as biosafety level 4 (BSL-4) agents. The arenavirus glycoprotein complex (GPC) contains three subunits—the retained stable-signal peptide (SSP), the receptor-binding subunit GP1, and the membrane fusion subunit GP2 (Lenz et al., 2001). Notably, the proximate external membrane region and TM of GP2, together with the ectodomain loop and TMs of SSP, form an SSP-GP2 interface, playing essential roles in regulating membrane fusion, and providing targets for distinct fusion inhibitors (Larson et al., 2008; Lee et al., 2008; York et al., 2008; York and Nunberg, 2009; Thomas et al., 2011; Burgeson et al., 2013a; Shankar et al., 2016; Wang et al., 2016; Wang et al., 2018).

Among these inhibitors, ST-161 is LASV specific (Burgeson et al., 2013a). In this study, we conducted structure-activity relationship (SAR) optimization of ST-161. As a result, 21 derivatives with IC_50_ values < 1 μmol/L are presented in Table S1. Hit compounds 21, 29 and 57 exhibiting robust inhibition of the LASV pseudotype virus (LASVpv, VSV backbone enveloped by LASV GPC with single cycle infection) entry with IC_50_ values lower than 0.2 nmol/L (Figs. 1A and S1), as well as hit compound 72 with an ester bond instead of acylhydrazone, were further investigated. To test whether the four hit compounds inhibit LASV entry by blocking the GPC-mediated membrane fusion, the inhibition effects of these compounds against LASV GPC mediated fusion were quantitatively determined by dual-luciferase assay (Thomas et al., 2011; Wang et al., 2018). Notably, the sequence of the inhibition effect obtained in this assay was 57, 21, 29, 72, which in line with the sequence specified in the LASVpv infection assay (Fig. 1B). Moreover, as the compounds were washed out before the low pH pulse, these findings suggest the hit compounds inhibited LASV entry by stabilizing the prefusion structure of GPC.

**Figure 1:**
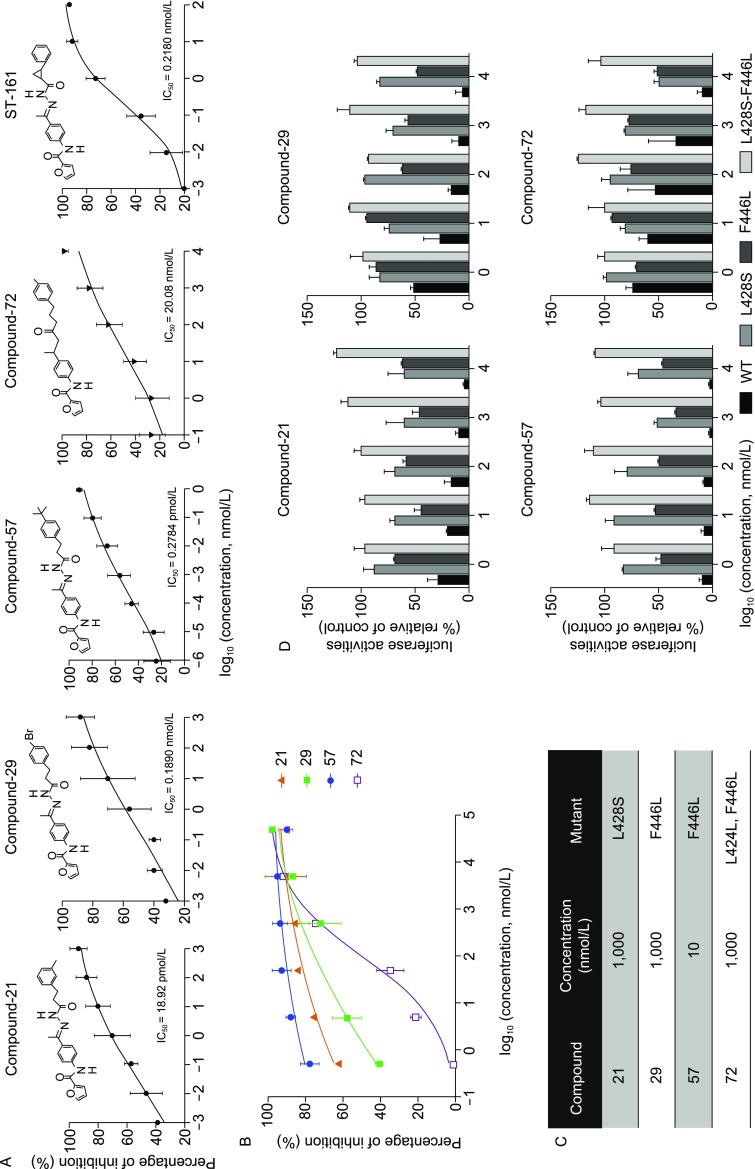

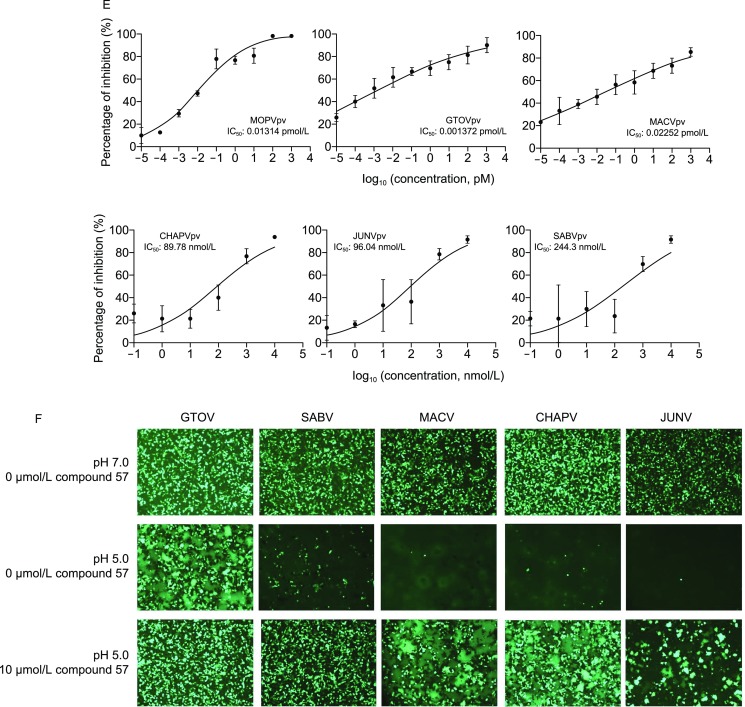
**Inhibitory effects of the four hit compounds**. (A) Dose-response curves of compounds 21, 29, 57, 72 and ST-161 for LASVpv infection of Vero cells as determined by measuring luciferase activities. Insets show the structure of each compound. (B) Hit compounds inhibited LASV GPC-mediated membrane fusion. Dual-luciferase assay was used to quantitatively evaluate the inhibitory activities of compounds against membrane fusion. Data are presented as means ± standard deviations (SD) for four independent experiments. (C) Selection of resistant LASVrv. The adaptive mutant selected by serially passaging LASVrv (MOI of 0.01) in the presence of each compound with indicated concentration. LASVrv passaging in vehicle served as a control in parallel. (D) Resistant and cross-resistant activities of the LASVpv with the adaptive mutants. Data are presented as means ± SD from three independent experiments. (E) Dose-response curves of compound 57 for inhibiting the pseudotype of MOPV and NW pathogenic arenaviruses infection. (F) Compound 57 inhibited NW pathogenic arenavirus GPC-mediated membrane fusion. Syncytium formation visualized using fluorescent microcopy after infection of 293T cells with pEGFP-N1 and pCAGGS-GTOV/SABV/MACV/CHAPV/JUNV GPC and treatment with compound 57. Images are representative fields from four or five independent experiments

To identify the viral target of the compounds, we selected adaptive mutant viruses by serially passaging the replication-competent recombinant virus of LASV (LASVrv, VSV backbone with a genome containing LASV GPC) in the presence of 1 μmol/L of any of the compounds 21, 29, and 72, or 10 nmol/L of compound 57, respectively, which approximately corresponded to the IC_90_ values of each compound. Parallel passaging of LASVrv in dimethyl sulfoxide (DMSO) was used as a control. As a result, two non-synonymous substitutions—L428S and F446L—were obtained in the compound 21 and the compound 29, 57 and 72 treatment groups, respectively (Fig. 1C). We next investigated the sensitivity of the two single nonsynonymous mutant viruses, as well as the double-mutant virus, to all the four hit compounds. Remarkably, the L428S mutant also conferred resistance to compounds 29, 57 and 72, in which L428S showed a stronger resistance to compound 57 compared with the F446L mutant. Moreover, the combined mutant virus was completely insensitive to any compound even at the highest tested concentration, suggesting these compounds might share the same viral target(s), and the adaptive mutants selected by similar compounds might show overlapping resistance effects (Fig. 1D).

Since the parent compound, ST-161, possessed specific antiviral activity against LASV, we investigated whether the four hit compounds extended their antiviral activities to other pathogenic arenaviruses. As shown in Figure S2, compounds 21, 29 and 72 largely maintained LASV specificity. In contrast, compound 57 showed promising inhibitory effects on the entry of NW pathogenic viruses, with a sharp blockage on the entry of GTOVpv and MACVpv in a picomolar range, as well as CHAPVpv, JUNVpv and SABVpv in a nanomolar range, suggesting that the tert-butyl (*t*-Bu) moiety in compound 57 might broaden the antiviral spectrum of the backbone (Fig. 1E). Notably, *t*-Bu was previously used to modify the acylhydrazone scaffold of ST-161 and led to a three- to twelvefold- decrease in IC_50_ value (Burgeson et al., 2013a), suggesting that this bulky, lipophilic moiety might raise the accessibility of the inhibitors to the viral target embedded in the TM domains. Meanwhile, the addition of the *t*-Bu motif might contribute to the specific contacts and result in a high binding affinity with the viral target. Notably, all four hit compounds had little effect on the entry of the OW pathogenic viruses, LCMVpv and LUJVpv. Further, none of the four hit compounds could inhibit the entry of EBOVpv and MARVpv (Fig. S2). Moreover, compound 57 blocked NW GPC-mediated membrane fusion. As shown in Figure 1F, when treated with a 15-min pulse of acidified (pH 5.0) medium, GPCs of GTOV, SABV, MACV, CHAPV and JUNV led to an extensive membrane fusion, resulting in the disappearance of the cell boundaries and the essentially black view which caused by the dilution of the green fluorescence. Since all the NW pathogenic arenaviruses utilize TfR1 as the cell receptor, we further investigated the impact of compound 57 on the virus-receptor interaction. We observed that compound 57 did not down-regulate the cell surface expression of TfR1, and it had no effect on binding of NW pathogenic viruses (Fig. S3). These results indicated that the extended antiviral activity of compound 57 acts intrinsically via targeting the membrane fusion process.

As the adaptive mutants, L428S and F446L, as well as the reported ST-193 sensitive residues, V431M and V435M (Larson et al., 2008), are located in the same side of the GP2 TM α-helix (Fig. 2A, sites d and a), we speculated that this side regulates the resistance to the fusion inhibitors. To address this, alanine scanning in the TM of LASV GP2 was carried out, and the sensitivity was assessed using compound 29 since it exerted a relatively mild inhibition, and thus made the effect more significant than the nanomolar fusion inhibitor such as compound 57. The TM domain examined in the current study started from L428, and extended to I452 because the proline at position 453 was thought to break the α-helix (Hastie et al., 2017). The fusion activities of the mutants are presented on the left column of each panel in Fig. 2C. Fusion activity was retained at all alanine substitutions except for D432A, in which little syncytium formation was observed. Further analysis revealed that D432A mutant had no effect on GPC maturational cleavage (Fig. 2C, inset panel). As the alanine mutant in the corresponding site of JUNV GPC (D424A) was a fusion-competence mutant (York et al., 2008; York and Nunberg, 2009), which was reported to lead a ~60% fusion, we reasoned this negative charged residue played different roles in LASV and JUNV GPCs mediate function.

**Figure 2:**
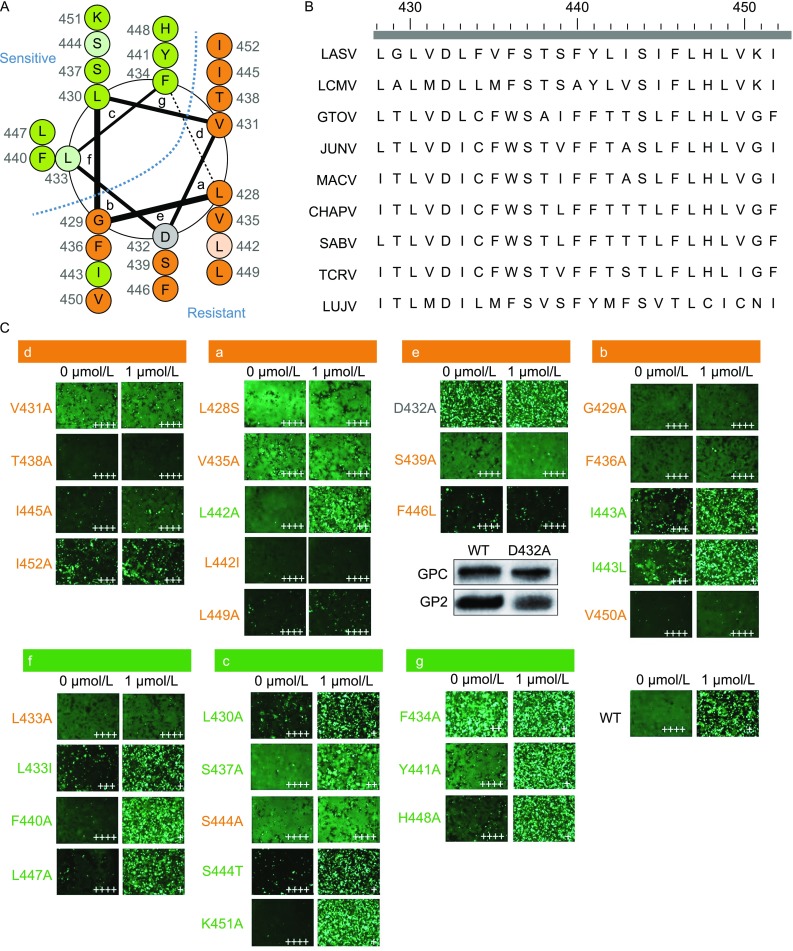
**Role of the TM of LASV GP2 in regulating sensitivity to compound 29**. (A) Helical-wheel project of the distinct sensitive (green) and resistant (orange) sides of TM of LASV GP2. The mutant failing in induce membrane fusion was labeled as gray. The mutants conferred their sensitivity and resistance in line with the side characteristic only when mutated to the similar residue were labeled as light green and light orange, respectively. The project was drawing by using DrawCoil 1.0 (Grigoryan, 2011). (B) Amino acid sequence alignment of the TM of arenaviruses GP2. (C) Mutations in the helix conferred sensitivity or resistance of LASV GPC to compound 29. Syncytium formation was visualized using fluorescent microcopy after infection of 293T cells with mutant pCAGGS-LASV GPC together with pEGFP-N1. Inset symbols showed the visual scoring of syncytium formation (“−” indicated no syncytia; “+, ++, +++, and ++++” indicated ~25%, ~50%, ~75% and complete syncytium formation, respectively). Images are representative fields from four to five independent experiments. The inset panel indicates that D432A mutant had no effect on the GPC maturational cleavage. The 293T cells transfected with WT and D432A GPC, respectively, were detected by Western blot using anti-LASV GP2 polyclonal antibody

As shown in Figure 2C, when treated with 1 µmol/L compound 29, those mutants caused a decrease in syncytium formation (that is, an increase of green puncta) were considered as the sensitive ones (green), while those unchanged mutants were judged as resistant ones (orange). Based on the distribution of those mutants, GP2 TM α-helix could be characterized as possessing distinct resistance (orange, sites d, a, b and e) and sensitive (green, sites f, c and g) sides (Fig. 2A). Of note, in the primary alanine scanning, L433A, L442A, I443A and S444A were found to act contrary to the resistance and sensitive side characteristics, in which L433A (site f) and S444A (site c) showed resistance to compound 29, while L442A (site a) and I443A (site b) were sensitive. To probe it, we individually mutated each residue into a similar residue. Remarkably, L433I and S444T rendered LASV GPC sensitive, while L442I conferred resistance to compound 29, which might be due to the effects of the similar side chains. I443L, however, maintained the sensitivity to compound 29. It has been reported that mutant A435I in JUNV GPC, the equivalent position to LASV I443, resulted in the resistance to fusion inhibitor ST-294 (York et al., 2008), suggesting that distinct fusion inhibitor might exclusively interact with special target(s) in the SSP-GP2 interface.

In this study, we conducted the SAR optimization of LASV specific fusion inhibitor ST-161, and found four hit compounds that retained the inhibitory effect against LASV GPC mediated membrane fusion, likely due to the effect on stabilization of the prefusion LASV GPC conformation (York et al., 2008; Thomas et al., 2011; Shankar et al., 2016). Especially, compound 57 could remarkably inhibit LASVpv infection at a picomolar range. Moreover, compound 57 extended its antiviral activities to NW pathogenic arenaviruses, in which the IC_50_ values of compound 57 against GTOVpv and MACVpv infection were three orders of magnitude less than those of ST-193 (Larson et al., 2008; Burgeson et al., 2013b; Dai et al., 2013), reaching a picomolar level. Selection and analysis of viruses resistant to the hit compounds revealed that the adaptive mutations were located in the transmembrane domain (TM) of GP2. Alanine substitution analysis indicated that one side of the GP2 TM helix regulates resistance to compound 29. Mutations in this side of the GPC made the virus resistant to compound 29, while mutations on the other side retained the sensitivity. Through our SAR study between the fusion inhibitor and GP2, we highlight the features involved in the regulation of sensitivity/resistance to fusion inhibitors and provide a platform for the design of entry inhibitors to combat arenavirus infections.

## Footnotes

We thank the The Center for Instrumental Analysis and Metrology and the Core Facility and Technical Support, Wuhan Institute of Virology for providing technical assistance.

This work was supported by the National Key Research and Development Program of China (2018YFA0507204), the National Natural Sciences Foundation of China (Grant No. 31670165), the Open Research Fund Program of CAS Key Laboratory of Special Pathogens and Biosafety, Wuhan Institute of Virology, and the Open Research Fund Program of Wuhan National Bio-Safety Level 4 Lab of CAS (NBL2017008), the Open Research Fund Program of the State Key Laboratory of Virology of China (2018IOV001).

Guangshun Zhang, Junyuan Cao, Yan Cai, Yang Liu, Yanli Li, Peilin Wang, Jiao Guo, Xiaoying Jia, Mengmeng Zhang, Gengfu Xiao, Yu Guo and Wei Wang declare that they have no conflict of interest.

## Electronic supplementary material

Below is the link to the electronic supplementary material.

Supplementary material 1 (PDF 819 kb)
