# Supplementary material for: Structure-activity relationship optimization for lassa virus fusion inhibitors targeting the transmembrane domain of GP2
Source: Protein Cell. 2019 Jan 10;10(2):137–42. doi: 10.1007/s13238-018-0604-x (PMC6340895; doi:10.1007/s13238-018-0604-x)
Supplement: Supplementary file 1 — Supplementary material 1 (PDF 819 kb) [file 13238_2018_604_MOESM1_ESM.pdf]

MATERIALS AND METHODS

Cells, plasmids, and viruses BHK-21, HEK 293T, Vero, and A549 cells were cultured in Dulbecco's modified Eagle's medium (HyClone, Logan, UT, USA) supplemented with 10% fetal bovine serum (FBS) (Gibco, Grand Island, NY, USA). The gene of LASV (Josiah strain, GenBank HQ688673.1), MOPV (GenBank AY772170.1), LCMV (Armstrong strain, GenBank AY847350.1), LUJV (GenBank NC_012776.1), GTOV (GenBank NC_005077.1), JUNV (XJ13 strain, GenBank NC_005081.1), MACV (Carvallo strain, GenBank NC_005078.1), SABV (GenBank U41071.1), CHAPV (GenBank NC_010562.1), EBOV (Mayinga strain, GenBank: EU224440.2), TCRV (GenBank NC_004293.1), and MARV (GenBank YP_001531156.1) were synthesized (Sangon Biotech, China) and subcloned into pCAGGS vector. Plasmids encoding each component of VSV proteins, i.e. pBS-N, pBS-P, pBS-L, pBS-G, were purchased from Kerafast. Plasmid pVSVΔG-eGFP (Plasmid #31842, Addgene) was modified into pVSVΔG-Rluc and pVSVΔG-eGFP-GPC for generating LASVpv and LASVrv, respectively. LASVpv was generated as described previously (Tani et al., 2010; Whitt, 2010; Zhang et al., 2016; Wang et al., 2018). Briefly, 293T cells transfected with pCAGGS-GPC were infected with pseudotype VSV carrying the luciferase gene at an MOI of 0.1 for 1 h. The culture supernatants were harvested and centrifuged 24 h later. LASVrv was generated by transfected BHK-21 cells in 6-wells plate, previously infected with a recombinant vaccinia virus (vTF7-3) encoding T7 RNA polymerase at a MOI of 5. After 45 min, cells were transfected with 11 μg of a mixture of plasmids with a

5:3:5:8:1 ratio of pVSVΔG-eGFP-GPC, pBS-N, pBS-P, pBS-G, and pBS-L (Geisbert et al., 2005; Safronetz et al., 2015). After 48 h, the supernatants were filtered to remove the vaccinia virus and inoculated into BHK-21 cells that had been transfected with pCAGGS-VSV G 24 h previously.

Synthesis of derivatives The derivatives were synthesized by our group (supplemental methods of synthesis of derivatives). Stock solutions of 20 mM were prepared with DMSO and diluted in DMEM with 2% FBS to different concentrations before use.

Inhibition of compounds against viral infection In the inhibition of pseudotype virus infection assay, Vero cells were seeded at a density of 1×10^4 cells per well in 96-well plates. After incubating overnight, cells were treated in triplicate with each compound; 1 h later, cells were infected with the pseudotype virus (MOI, 0.01), and the supernatant was removed 1 h post-infection. The infected cells were lysed 23 h later, and luciferase activity was measured using the *Renilla* luciferase (Rluc) assay system (Promega, Madison, WI).

In the inhibition of LASVrv infection assay, A549 cells were seeded at a density of 1.2×10^4 cells per well in 96-well plates. After incubating overnight, cells were treated in duplicate with each compound; 1 h later, LASVrv with MOI of 0.1 were added to the cells. After 24 h, cells were fixed with 4 % paraformaldehyde and stained with DAPI (Sigma-Aldrich). The images were recorded with the Operetta high-content imaging system (PerkinElmer).

The cell viability was determined by using

3-(4,5-dimethyl-2-thiazolyl)-2,5-diphenyl-2H-tetrazolium bromide (MTT) assay (Wang et al., 2018). The IC_{50} and CC_{50} values were calculated using GraphPad Prism 6.

Selection of adaptive mutants Drug-resistant viruses were generated by passaging LASVrv on Vero cells in the presence of each compound. LASVrv passaged in parallel in the presence of 0.5% DMSO acted as a control. After three rounds of passaging, RNA was extracted by TRIzol (Invitrogen) and reverse transcribed by using the PrimeScript RT reagent kit (TaKaRa). The GPC segment was amplified and sequenced as previously described (Wang et al., 2018). Mutant sites were introduced into LASVpv, and the compound sensitivities were determined using the Rluc assay.

Membrane fusion assay The 293T cells transfected with pEGFP-N1 in combination with pCAGGS-LASV GPC or the empty pCAGGS were treated with compound or vehicle (DMSO) for 1 h, followed by incubation for 15 min with acidified (pH 5.0) medium. The cells were then placed in neutral medium, and syncytium formation was visualized 3 h later via fluorescent microscopy.

For quantification of the luciferase-based fusion assay, 293T cells in 24-well plate transfected with both pCAGGS-LASV GPC (0.25 μg) and plasmids expressing T7 RNA polymerase (pCAGT7, 0.25 μg) were co-cultured at a ratio of 3:1 with targeted cells transfected with pT7EMCVLuc (2 μg per well for 6-wells plate) and 0.1 μg pRL-CMV (plasmids used in this assay were kindly provided by Yoshiharu Matsuura, Osaka University, Osaka, Japan). After 12 h of incubation, the compound treatment and low pH induction were conducted as described above. Cell fusion activity was

quantitatively determined after 24 h by measuring firefly luciferase activity expressed by pT7EMCVLuc and was standardized with Rluc activity expressed by pRL-CMV using the Dual-Glo luciferase assay (Promega) (Takikawa et al., 2000; Thomas et al., 2011; Wang et al., 2018).

Western blotting Equal amounts of cell lysates were subjected to SDS-PAGE and transferred onto a polyvinylidene difluoride (PVDF) membrane (Millipore). TfR1 and GAPDH were detected using anti-TfR1 monoclonal antibody (#13113, Cell Signaling) and anti-GAPDH monoclonal antibody (60004-1-Ig, Proteintech), respectively. LASV GPC and GP2 were detected using rabbit anti-GP2 polyclonal antibody (1:1000 dilution) prepared by our group following reported methods (Eschli et al., 2006).

Binding assay A549 cells were preincubated with compound 57 or vehicle at 37 ℃ for 1 h, followed by incubation with the pseudotype of viruses with MOI of 0.5 in the presence or absence of compound at 4 ℃ for an additional 1 h. After extensively washing thrice with cold phosphate-buffered saline (PBS), the bound virus was quantified via reverse transcriptase quantitative PCR (RT-qPCR) (Wang et al., 2018).

Eschli, B., Quirin, K., Wepf, A., Weber, J., Zinkernagel, R., and Hengartner, H. (2006). Identification of an N-terminal trimeric coiled-coil core within arenavirus glycoprotein 2 permits assignment to class I viral fusion proteins. J Virol 80, 5897-5907.

Geisbert, T.W., Jones, S., Fritz, E.A., Shurtleff, A.C., Geisbert, J.B., Liebscher, R., Grolla, A., Stroher, U., Fernando, L., Daddario, K.M.*, et al.* (2005). Development of a new vaccine for the prevention of Lassa fever. PLoS Med 2, e183.

Safronetz, D., Mire, C., Rosenke, K., Feldmann, F., Haddock, E., Geisbert, T., and Feldmann, H. (2015). A recombinant vesicular stomatitis virus-based Lassa fever vaccine protects guinea pigs and macaques against challenge with geographically and genetically distinct Lassa viruses. PLoS Negl Trop Dis 9, e0003736.

Takikawa, S., Ishii, K., Aizaki, H., Suzuki, T., Asakura, H., Matsuura, Y., and Miyamura, T. (2000). Cell fusion activity of hepatitis C virus envelope proteins. J Virol 74, 5066-5074.

Tani, H., Shiokawa, M., Kaname, Y., Kambara, H., Mori, Y., Abe, T., Moriishi, K., and Matsuura, Y. (2010). Involvement of ceramide in the propagation of Japanese encephalitis virus. J Virol 84, 2798-2807.

Thomas, C.J., Casquilho-Gray, H.E., York, J., DeCamp, D.L., Dai, D., Petrilli, E.B., Boger, D.L., Slayden, R.A., Amberg, S.M., Sprang, S.R., *et al.* (2011). A specific interaction of small molecule entry inhibitors with the envelope glycoprotein complex of the Junin hemorrhagic fever arenavirus. J Biol Chem 286, 6192-6200.

Wang, P., Liu, Y., Zhang, G., Wang, S., Guo, J., Cao, J., Jia, X., Zhang, L., Xiao, G., and Wang, W. (2018). Screening and Identification of Lassa Virus Entry Inhibitors from an FDA-Approved Drugs Library. J Virol 92, e00954-00918.

Whitt, M.A. (2010). Generation of VSV pseudotypes using recombinant DeltaG-VSV for studies on virus entry, identification of entry inhibitors, and immune responses to vaccines. J Virol Methods 169, 365-374.

Zhang, L.K., Xin, Q.L., Zhu, S.L., Wan, W.W., Wang, W., and Xiao, G. (2016). Activation of the RLR/MAVS signaling pathway by the L protein of Mopeia virus. J Virol 90, 10259-10270.

SUPPLEMENTARY FIGURES

Figure S1. Inhibitory effects of the four hit compounds against LASVpv infection.

(A) High-content images showing the GFP (green) and nuclei (blue) of A549 cells, treated with each compound and later infected with LASVrv (MOI of 0.1) stained with DAPI. (B) The effects of the four hit compounds on VSVpv infection.

Figure S2. Broad-spectrum antiviral activity of the hit compounds against different arenaviruses and filoviruses.

(A) Antiviral activities of compound 21, 29 and 72 for infection by pseudotype of GTOV, JUNV, MACV, SABV, CHAPV, LCMV, LUJV, MOPV, EBOV, and MARV (added at an MOI of 0.01). The effects were assessed by measuring luciferase activity. Data are presented as means ± SD from two independent experiments. (B) The effect of compound 57 on LUJVpv, LCMVpv, EBOVpv and MARVpv infection.

Figure S3. Effects of compound 57 on pseudotype virus cell binding.

(A) Effects of compound 57 on cell expression of TfR1. A549 cells were incubated with compound 57 or vehicle at 37℃ for 2 h and then analyzed by western blotting for TfR1 and GAPDH. (B) Effects of compound 57 on the pseudotype of NW pathogenic arenaviruses binding. A549 cells were pre-incubated with compound 57 and then incubated with the pseudotype of indicated viruses (MOI, 0.5) for 1 h. The bound virus was later quantified via RT-qPCR.

Figure S1.

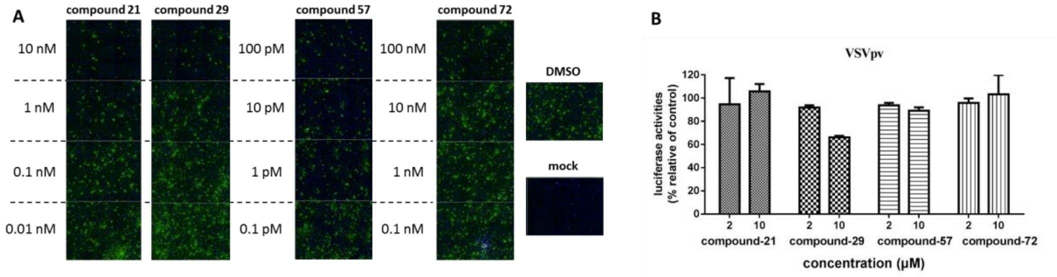

A
compound 21
compound 29
compound 57
compound 72
10 nM
1 nM
0.1 nM
0.01 nM
100 pM
10 pM
1 pM
0.1 pM
100 nM
10 nM
1 nM
0.1 nM
DMSO
mock
B
VSVpv
luciferase activities (% relative of control)
120
100
80
60
40
20
0
2
10
compound-21
compound-29
compound-57
compound-72
concentration (µM)

Figure S2.

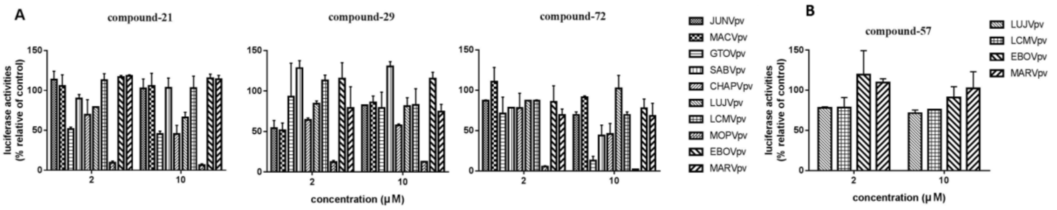

Figure S3.

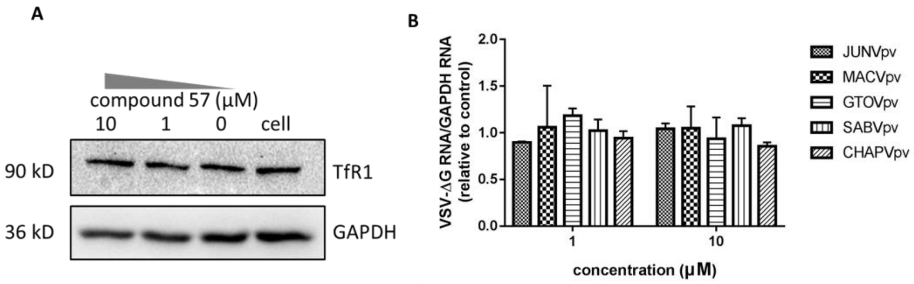
A
compound 57 (μM)
10
1
0
cell
90 kD
TfR1
36 kD
GAPDH
B
VSV-ΔG RNA/GAPDH RNA (relative to control)
2.0
1.5
1.0
0.5
0.0
1
10
concentration (μM)
JUNVpv
MACVpv
GTOVpv
SABVpv
CHAPVpv

Table 1 Effects of R substitutions

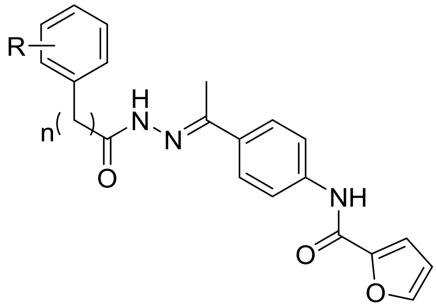

compound	R	n	SMILES	IC$_{50}$ (nM)	CC$_{50}$ (μM)
3	H	2	O=C(CCC1=CC=CC=C1)N/N=C(C)/C2=CC=C(NC(C3=CC=CO3)=O)C=C2	868	>30
7	H	1	O=C(CC1=CC=CC=C1)N/N=C(C)/C2=CC=C(NC(C3=CC=CO3)=O)C=C2	309	>30
8	*p*-, *o*-Cl	1	O=C(CC1=C(Cl)C=C(Cl)C=C1)N/N=C(C)/C2=CC=C(NC(C3=CC=CO3)=O)C=C2	185	>30
9	*p*-CH_3	2	O=C(CCC1=CC=C(C)C=C1)N/N=C(C)/C2=CC=C(NC(C3=CC=CO3)=O)C=C2	0.37	>30
10	*o*-F	2	O=C(CCC1=C(F)C=CC=C1)N/N=C(C)/C2=CC=C(NC(C3=CC=CO3)=O)C=C2	3.0	>30
20	*o*-CH_3	2	O=C(CCC1=C(C)C=CC=C1)N/N=C(C)/C2=CC=C(NC(C3=CC=CO3)=O)C=C2	0.92	>30
21	*m*-CH_3	2	O=C(CCC1=CC(C)=CC=C1)N/N=C(C)/C2=CC=C(NC(C3=CC=CO3)=O)C=C2	0.019	>30
22	*p*-O-CH_3	2	O=C(CCC1=CC=C(OC)C=C1)N/N=C(C)/C2=CC=C(NC(C3=CC=CO3)=O)C=C2	0.050	>30
23	*p*-CH_3	3	O=C(CCCC1=CC=C(C	0.30	>30

			)C=C1)N/N=C(C)/C2=CC=C(NC(C3=CC=CO3)=O)C=C2		
24	*p*-NO_2	2	O=C(CCC1=CC=C([N+]([O-])=O)C=C1)N/N=C(C)/C2=CC=C(NC(C3=CC=CO3)=O)C=C2	100	>30
25	*m*-NO_2	2	O=C(CCC1=CC([N+]([O-])=O)=CC=C1)N/N=C(C)/C2=CC=C(NC(C3=CC=CO3)=O)C=C2	94	>30
26	*p*-F	2	O=C(CCC1=CC=C(F)C=C1)N/N=C(C)/C2=CC=C(NC(C3=CC=CO3)=O)C=C2	1.9	>30
27	H	4	O=C(CCCCC1=CC=CC=C1)N/N=C(C)/C2=CC=C(NC(C3=CC=CO3)=O)C=C2	1.7	>30
28	*p*-, *o*-O-CH_3	2	O=C(CCC1=C(OC)C=C(OC)C=C1)N/N=C(C)/C2=CC=C(NC(C3=CC=CO3)=O)C=C2	93	>30
29	*p*-Br	2	O=C(CCC1=CC=C(Br)C=C1)N/N=C(C)/C2=CC=C(NC(C3=CC=CO3)=O)C=C2	0.19	>30
30	*o*-CF_3	2	O=C(CCC1=C(C(F)(F)F)C=CC=C1)N/N=C(C)/C2=CC=C(NC(C3=CC=CO3)=O)C=C2	2.7	>30
54	*p*-O-CH_3	1	O=C(CC1=CC=C(OC)C=C1)N/N=C(C)/C2=CC=C(NC(C3=CC=CO3)=O)C=C2	200	>30
56	H	3	O=C(CCCC1=CC=CC=C1)N/N=C(C)/C2=CC=C(NC(C3=CC=CO3)=O)C=C2	264	>30
57	*p*-^{t}Bu	2	O=C(CCC1=CC=C(C(C)(C)C)C=C1)N/N=C(C)/C2=CC=C(NC(C3=CC=CO3)=O)C=C2	< 0.001	>30

71	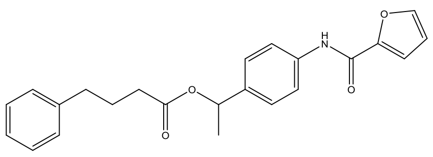	O=C(OC(C)C1=CC=C(NC(C2=CC=CO2)=O)C=C1)CCCC3=CC=CC=C3	189	>30
72	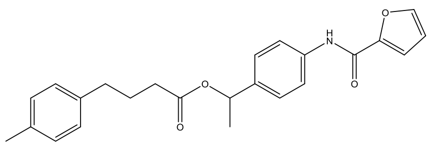	O=C(OC(C)C1=CC=C(NC(C2=CC=CO2)=O)C=C1)CCCC3=CC=C(C)C=C3	22	>30

Supplemental material 1

Synthesis of LASVI-57

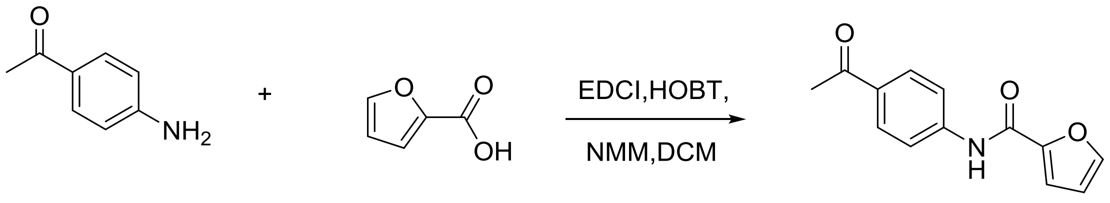

01

Firstly, 2-furoic acid (1.7 g, 14.8 mmol) and 2 mL N-methylmorpholine (NMM) were mixed with 15 mL dry dichloromethane (DCM), followed by the addition of *N*-(3-dimethylaminopropyl)-*N*-ethylcarbodiimide hydrochloride (EDCI; 2.1 g, 11.0 mmol) and 1-hydroxy benzotriazole (HOBT; 1.5 g, 11.0 mmol), and the mixture was stirred for 20 min. Next, 4-aminoacetophenone (1.0 g, 7.4 mmol) was added and the resulting solution was stirred overnight. The reaction mass was washed with 1 M HCl aq, followed by saturation with $NaHCO_3$ aq. The organic layer was collected and dried with anhydrous Na_2SO_4. The DCM was removed using a rotary evaporator, the resulting mass was purified by silica gel chromatography. Around 1.2 g of compound **01** was obtained (white solid, 70% yield). ^{1}H NMR (600 MHz, Chloroform-d) δ 8.30 (s, 1H), 8.00 (d, J = 8.3 Hz, 2H), 7.79 (d, J = 8.3 Hz, 2H), 7.55 (s, 1H), 7.19 – 7.36 (m, 1H), 6.49 – 6.66 (m, 1H), 2.61 (s, 3H). ESI-MS: calculated for C13H11NO3 [M+H]+: 230.24, found 230.10.

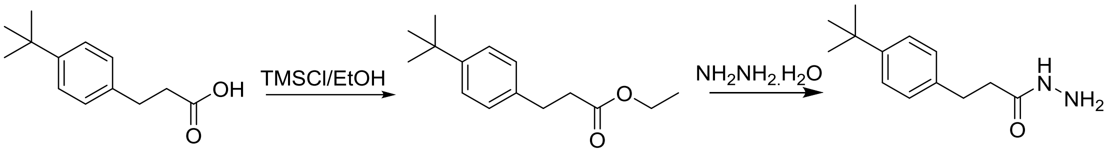

02 **03**

3-(4-tert-butyl-phenyl)-propionic acid (0.3 g, 1.5 mmol) and trimethylsilyl chloride (TMSCl; 0.56 mL, 4.5 mmol) was mixed with 5 mL ethanol, and the resulting solution was heated at 75 ℃. After the completion of reaction, monitored by TLC, ethanol was removed by a rotary evaporator under vacuum, and the resulting mass was diluted with ethyl acetate (EA), and washed with water twice. The EA phase was collected and dried with anhydrous Na_2SO_4. EA was removed under vacuum to obtain 0.3 g of compound **02**, which could be used directly without further purification. Compound **02** was dissolved in 5 mL ethanol, followed by the addition of 1.4 mL 80％ $H_2NNH_2.H_2O$ (22.5 mmol), and the resulting solution was heated at 75 ℃ for 3. After the completion of reaction, the ethanol was removed by rotary evaporator and the resulting mass was purified by silica gel chromatography to obtain 0.2 g of compound **03** (white solid, 70% yield). ^{1}H NMR (600 MHz, Chloroform-*d*) δ 7.30 – 7.37 (m, 2H), 7.10 – 7.17 (m, 2H), 6.68 – 6.70 (m, 1H), 3.90 (s, 2H), 2.96 (dd, *J* = 8.6, 7.0 Hz, 2H), 2.47 (dd, *J* = 8.5, 7.0 Hz, 2H), 1.33 (s, 9H). ESI-MS: calculated for $C_{13}H_{20}N_2O$ [M+H]$^+$: 221.32, found 221.37.

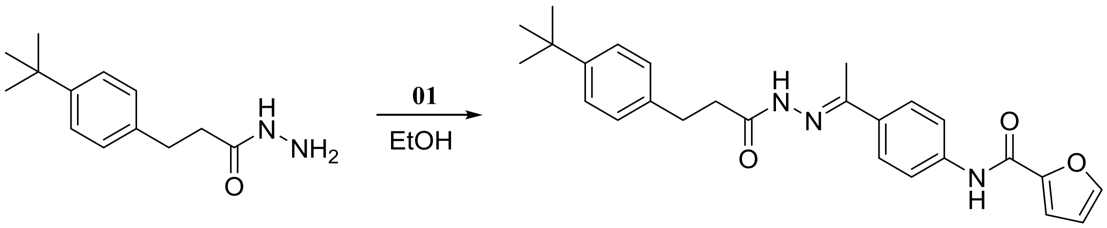

03 **LASVI-57**

Compound **03** (0.1 g, 0.5 mmol) and compound **01** (0.1 g, 0.6 mmol) was mixed with 2 mL ethanol and 30 μL acetic acid (AcOH). The resulting mass was heated at

75 ℃ for 5 h, during which the mixture turned clear. After the completion of reaction, the ethanol was removed by rotary evaporator and the resulting mass was purified by silica gel chromatography to obtain 0.2 g LASVI-57 as a white solid (93% yield). ^{1}H NMR (600 MHz, DMSO-d_6) δ 10.24 – 10.45 (m, 2H), 7.96 (s,1H), 7.70 – 7.84 (m, 4H), 7.37 (dd, *J* = 8.0, 3.5 Hz, 1H), 7.30 (d, *J* = 7.5 Hz, 2H), 7.19 (t, *J* = 7.9 Hz, 2H), 6.69 – 6.74 (m, 1H), 2.54 – 3.00 (m, 4H), 2.22 (s, 3H), 1.26 (d, *J* = 2.9 Hz, 9H). ^{13}C NMR (151 MHz, DMSO-d_6) δ 174.72, 168.76, 156.65, 151.20, 147.91, 147.18, 146.26, 139.05, 133.93, 128.40, 126.99, 125.45, 120.25, 115.36, 112.63, 35.41, 34.50, 31.66, 30.47, 14.03. ESI-MS: calculated for $C_{26}H_{29}N_3O_3$ [M+H]$^+$: 432.54, found 432.60.

Synthesis of LASVI-21 and LASVI-29:

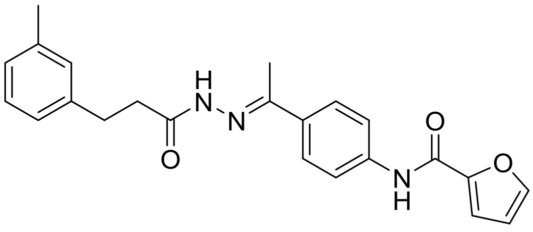

LASVI-21

LASVI-21 was synthesized following the similar protocol of synthesis of LASVI-57. ^{1}H NMR (600 MHz, DMSO-d_6) δ 10.23 – 10.43 (m, 2H), 7.95 (d, *J* = 1.7 Hz, 1H), 7.69 – 7.84 (m, 4H), 7.36 (dd, *J* = 7.6, 3.5 Hz, 1H), 7.17 (q, *J* = 7.6 Hz, 1H), 7.03 – 7.11 (m, 2H), 7.00 (t, *J* = 7.3 Hz, 1H), 6.72 (dd, *J* = 3.5, 1.7 Hz, 1H), 2.57 – 3.01 (m, 4H), 2.28 (d, *J* = 6.7 Hz, 3H), 2.22 (s, 3H). ^{13}C NMR (151 MHz, DMSO-d_6) δ 174.72, 168.75, 156.66, 151.20, 147.88, 147.19, 146.29, 140.78, 137.71, 133.92, 129.48, 128.64,126.99, 125.79, 120.25, 115.38, 112.65, 35.46, 30.99, 21.50, 14.03. ESI-MS: calculated for $C_{23}H_{23}N_3O_3$ [M+H]$^+$: 390.46, found 390.62.

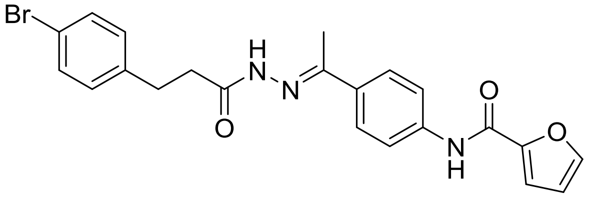

LASVI-29

LASVI-29 was synthesized following the similar protocol of synthesis of LASVI-57. ^{1}H NMR (600 MHz,

DMSO-d6) δ 10.25 – 10.44 (m, 2H), 7.92 – 7.96 (m, 1H), 7.69 – 7.83 (m, 4H), 7.41 – 7.52 (m, 2H), 7.32 – 7.39 (m, 1H), 7.24 (dd, J = 9.9, 8.2 Hz, 2H), 6.72 (dd, J = 3.5, 1.7 Hz, 1H), 2.59 – 3.01 (m, 4H), 2.21 (d, J = 2.1 Hz, 3H). 13C NMR (151 MHz, DMSO-d6) δ 174.45, 168.49, 156.65, 151.26, 147.88, 147.32, 146.29, 140.57, 133.89, 131.55, 131.11, 127.01, 120.26, 115.38, 112.65, 35.03, 30.27, 14.05. ESI-MS: calculated for $C_{22}H_{20}BrN_3O_3$ [M+H]$^+$: 455.31, found 455.40.

Synthesis of LASVI-72

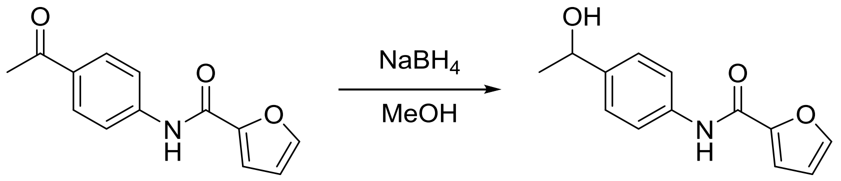

01 **04**

Compound **01** (1.0 g, 4.4 mmol) was dissolved in 15 mL methanol, $NaBH_4$ (0.4 g, 9.5 mmol) was added portion-wise at 0 ℃; the reaction mixture was then stirred for 3 h at 0 ℃. After the completion of reaction, the pH was adjusted to 5 by addition of 1 M HCl aq, then methanol was removed under vacuum and the resulting mass was dissolved in EA. The organic solution was washed twice with water and dried with anhydrous Na_2SO_4, The EA was removed by rotary evaporator under vacuum and the resulting mass was purified by silica gel chromatography which yielded 1.0 g of compound **04** (light yellow oil, 99% yield).

^{1}H NMR (400 MHz, Chloroform-d) δ 8.18 – 8.04 (m,1H), 7.68 – 7.60 (m, 2H), 7.54 (dd, J = 1.8, 0.8 Hz,1H), 7.43 – 7.35 (m, 2H), 7.26 (dd, J = 3.5, 0.8 Hz, 1H), 6.59 (dd, J = 3.5, 1.8 Hz, 1H), 4.92 (q, J = 6.5 Hz, 1H), 1.88 (s, 1H), 1.52 (d, J = 6.5 Hz, 3H). ESI-MS: calculated for C13H13NO3 [M+H]$^+$: 232.25, found 232.20.

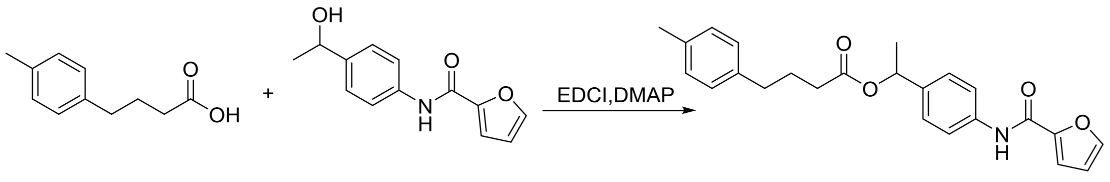

04 LASVI-72

A 50 mL round bottom flask was charged with 3 mL DCM, 4-(p-tolyl)butyric acid (0.2 g, 1.0 mmol), compound 04 (0.2 g，0.7 mmol), EDCI (0.6 g，3.2 mmol) and DMAP (0.4 g，3.2 mmol). The resulting mixture was stirred at ambient temperature overnight, after which the reaction mixture was washed with 1 M HCl aq and subsequently saturated with $NaHCO_3$ aq. The organic phase was collected and dried with anhydrous Na_2SO_4, The DCM was removed by rotary evaporator under vacuum, and the resulting mass was purified by silica gel chromatography, which yielded 0.2 g of LASVI-72 (light yellow oil, 83% yield).^{1}H NMR (600 MHz, Chloroform-*d*) δ 8.17 (d, *J* = 10.6 Hz, 1H), 7.67 (dd, *J* = 8.6, 1.9 Hz, 2H), 7.52 (d, *J* = 2.1 Hz, 1H), 7.39 – 7.35 (m, 2H), 7.26 (dd, *J* = 3.5, 0.9 Hz, 1H), 7.11 (d, *J* = 7.8 Hz, 2H), 7.07 (d, *J* = 8.0 Hz, 2H), 6.58 (dd, *J* = 3.5, 1.8 Hz, 1H), 5.91 (q, *J* = 6.6 Hz, 1H), 2.62 (t, *J* = 7.6 Hz, 2H), 2.40 – 2.31 (m, 5H), 1.96 (p, *J* = 7.5 Hz, 2H), 1.56 (d, *J* = 6.7 Hz, 3H). ^{13}C NMR (151 MHz, Chloroform-*d*) δ 172.75, 156.05, 147.78, 144.22, 138.30, 137.90, 137.01, 135.40, 129.07, 128.37, 127.00, 119.98, 115.33, 112.63, 71.76, 34.66, 33.94, 26.65, 22.10, 21.00. ESI-MS: calculated for $C_{24}H_{25}NO_4$ [M+H]$^+$: 392.47, found 392.54.
